# Angiogenic Potential in Biological Hydrogels

**DOI:** 10.3390/biomedicines8100436

**Published:** 2020-10-20

**Authors:** Maria Vittoria Giraudo, Dalila Di Francesco, Marta Calvo Catoira, Diego Cotella, Luca Fusaro, Francesca Boccafoschi

**Affiliations:** 1 Department of Health Sciences, University of Piemonte Orientale, 28100 Novara, Italy; 20023156@studenti.uniupo.it (M.V.G.); 20019023@studenti.uniupo.it (D.D.F.); diego.cotella@med.uniupo.it (D.C.); 2 Tissuegraft srl, 28100 Novara, Italy; marta.catoira@uniupo.it (M.C.C.); luca.fusaro@med.uniupo.it (L.F.)

**Keywords:** hydrogel, natural polymers, decellularized matrix

## Abstract

Hydrogels are three-dimensional (3D) materials able to absorb and retain water in large amounts while maintaining their structural stability. Due to their considerable biocompatibility and similarity with the body’s tissues, hydrogels are one of the most promising groups of biomaterials. The main application of these hydrogels is in regenerative medicine, in which they allow the formation of an environment suitable for cell differentiation and growth. Deriving from these hydrogels, it is, therefore, possible to obtain bioactive materials that can regenerate tissues. Because vessels guarantee the right amount of oxygen and nutrients but also assure the elimination of waste products, angiogenesis is one of the processes at the base of the regeneration of a tissue. On the other hand, it is a very complex mechanism and the parameters to consider are several. Indeed, the factors and the cells involved in this process are numerous and, for this reason, it has been a challenge to recreate a biomaterial able to adequately sustain the angiogenic process. However, in this review the focal point is the application of natural hydrogels in angiogenesis enhancing and their potential to guide this process.

## 1. Angiogenesis Process and the Key role in Tissue Regeneration

Angiogenesis is the neo-formation of new blood vessels from pre-existing vessels [1]. As the bloodstream is an essential component for the dispersion of metabolites and nutrients and to remove the excess of toxic products, these processes are fundamental for the proper functioning of the tissues. In fact, the process itself and consequently the capillary structure, can be modified by changing the cells’ metabolic activity. Moreover, the coordination and viability of vessels walls are regulated by oxygen and haemodynamic factors [2].

Throughout the years, the inhibition and stimulation of angiogenesis have been the focus of numerous studies as it was demonstrated that the alteration of its normal function induces several diseases. However, angiogenesis enhancing can be therapeutic, for instance in ischemic heart disease, wound healing, and peripheral arterial disease [1].

In contrast to the term substitution, regeneration means “to restore the physical integrity of cells, tissues and organs by means of the organisms’ own repair mechanisms” [2]. To pursue this goal, biomaterials, defined as substances with the capability to replace organs or tissues, either cure or expand them [3], are a promising group of materials due to their biodegradability and biocompatibility. Other properties such as the non-toxicity and the ability to sustain a sterilization process also characterize this type of materials. Due to the several applications such as drug delivery, cell encapsulation, tissue engineering scaffolds, wound dressing, soft tissue replacement, contact lenses, and biosensor, hydrogels can be one of the most promising biomaterials in the biomedical field [3,4].

Especially, the regeneration of a tissue can develop only with a simultaneous progress of the vessel system. In this context, angiogenesis is the crucial process because it defines the sprouting of a vascular system based on pre-existing capillaries via endothelial cells migration and proliferation. The process involves a dynamic crosstalk between the surrounding tissue and different cell phenotypes such as endothelial cells, macrophages, pericytes, monocytes, fibroblasts, and smooth muscle cells [2].

The angiogenic process consists of sequential steps [5]:

- vasodilatation of the original vessels, in response to nitrogen monoxide and to the increasing permeability triggered by vascular endothelial growth factor (VEGF);
- separation of the pericytes from the luminal surface and the break of basement membrane thus allowing the formation of the new vessel:
- migration and proliferation of the endothelial cells to the damaged tissue;
- neo-formation of vessel lumen;
- recruitment of the peri-endothelial cells (pericytes for small capillary and smooth muscle cells for larger vessels);
- inhibition of endothelial cell proliferation;
- deposition of the basement membrane and initiating of blood flow.

The angiogenic process involves the extracellular matrix (ECM), cell-cell interactions, many signaling pathways, and enzymes. Here following a short description of their own roles.

## 2. Extracellular Matrix

Proteins, proteoglycans, glycoproteins, and polysaccharides are the main components of the ECM. The interactions between the ECM components and cell receptors allow the creation of a complex network [6,7].

Especially, during the angiogenic process the ECM structure and composition change and these transformations play a critical role in determining the regulation of new blood vessels’ growth. An important alteration involves the ECM enzymes produced by the endothelial cells and stimulated by VEGF, fibroblast growth factors (FGF), and transforming growth factor beta (TGF-β). The metalloproteinases of the matrix (MMPs) which are part of the ECM enzymes, degrade the ECM to remodel and extend the vascular structure. There are five groups of MMPs and they are produced as pro-enzymes which must be proteolytically processed to be activated. The zinc that is present in the endopeptidases, is the site where other enzymes can disrupt the MMPs to activate them [5,8].

Another important change concerns the production by the endothelial cells of neo-synthetized ECM molecules such as collagen, fibronectin, and glycosaminoglycans (GAGs) with the function to modulate the adhesion of the cells through integrins [7,9]. The integrin receptors are the main regulators in the early part of angiogenesis because they potentiate the signaling events. When they are activated by growth factors (GFs), they transduce the signal through the Rho kinase and the family of Src kinases, and which facilitate the retraction of endothelial cells. It this way, there will be enough space to let platelets passing to the lamina that underlies the endothelial cell margin and releasing the content of the α-granules, such as protease and lipase to the ECM and angiogenesis-regulating factors like VEGF, PDGF and Sphingosine-1-phosphate (S1P) [9].

## 3. Growth Factors

**Figure 1:**
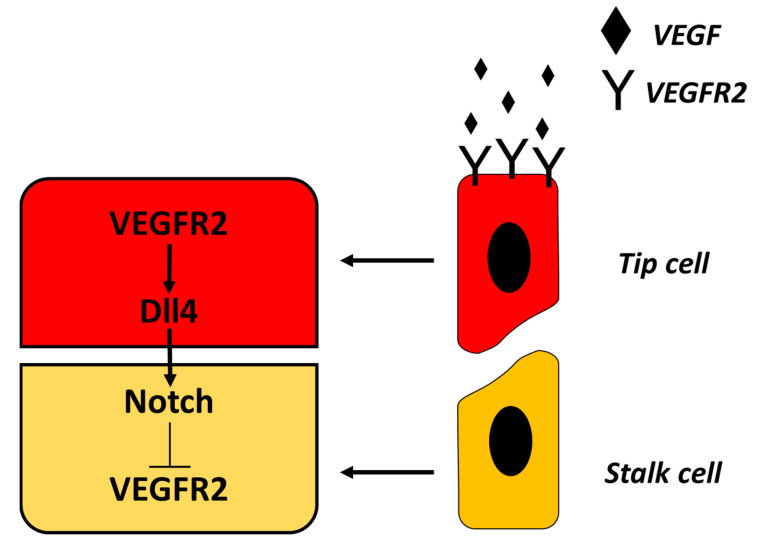
Current model of VEGF-Notch signaling pathway.

VEGFs, mainly VEGF-A, stimulate the migration and the proliferation of the endothelial cells, initiating the capillary sprouting process of angiogenesis. It also induces vasodilation, stimulating the production of NO and contributes to create a new vascular lumen [10]. Most of the types of parenchymal cells secrete this key proangiogenic growth factor that can anchor VEGF-A receptors (VEGFR2) expressed on the membrane of the endothelial cells. Through different signaling pathways, the interaction receptor-substratum induces proliferation, survival, permeability, and migration. Fibroblast growth factors (FGF), mainly FGF-2, stimulate the proliferation of the endothelial cells and also promote macrophages and fibroblasts migration to the damaged tissue to heal the epidermal wounds. Angiopoietin 1 and 2 are growth factors important for their role in the structure maturation of the new vessels that require a stabilization with pericytes and smooth muscle cells. Other factors, like the transforming growth factor beta (TGF-β) and the platelet-derived growth factor (PDGF) are also important for the stabilization of angiopoietin 1 and 2: PDGF recruits the smooth muscle cells and TGF-β inhibits the endothelial proliferation and migration and increases the proteins production from the ECM [1,5]. Thanks to the crosstalk between VEGF and Notch signaling, the signaling pathway activated regulates the sprouting and ramification of the new vessels. It assures that vasculogenesis has the adequate space to carry the right amount of blood for the damage tissue. The Notch signaling pathway exerts multiple roles during vascular development and physiology in vertebrates. It is represented by a large family of receptors. In mammals, four Notch family receptors (NOTCH1-4) have been studied and they are composed by an extracellular domain which interacts with ligands that are also single-pass type I transmembrane proteins [11]. The signal activated by ligand binding is conducted intracellularly by a process involving proteolytic cleavage of the receptor, followed by the nuclear translocation of the intracellular domain of the Notch family protein. The signal causes modifications that are included in the regulation and differentiation of endothelial cells and vascular smooth muscle cells, and in the regulation of blood vessel sprouting and branching during angiogenesis [5]. Figure 1 represents the current model of VEGF-Notch signaling pathways and interactions. In particular VEGF binding to its receptor on tip cells leads to the activation of Notch signaling in stalk cells through its ligand Delta-like ligand 4, or Dll4, which in turn down regulates the VEGF receptor 2, therefore regulating tip cell sprouting [12]. However new studies reevaluate the influence of Notch signaling in new vessel formation [13,14], these would allow for a better understanding of angiogenesis, which can be exploited to optimize the performance of new biomaterials.

## 4. Natural Hydrogels

The term “hydrogel” indicates 3D network structures obtained from a class of synthetic and/or natural polymers which can absorb and retain significant amounts of water. The hydrogel structure is formed by hydrophilic groups or domains present in a polymeric network upon the hydration in an aqueous environment [15].

**Figure 2:**
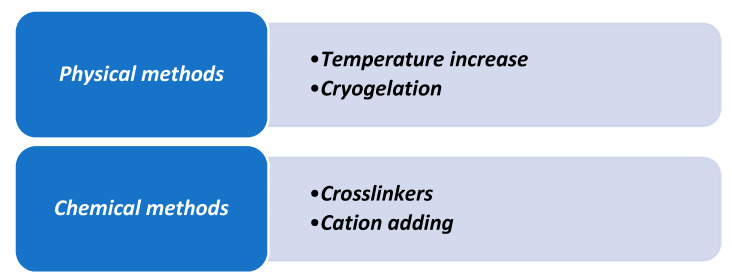
Gelation agents in natural hydrogels.

The integrity of the 3D structure of hydrogels in their swollen state is maintained either by physical or chemical crosslinking. The type of crosslink can be the criteria to classify hydrogels, but they can also be classified based on their methods of preparation, the ionic charges, the sources, the nature of swelling with changes in the environment, and the rate of biodegradation [3,16]. Understanding the mechanism of hydrogels network formation allows to optimize their physical characteristic for specific application. Different techniques can be used to replicate the transition from a liquid state to a gel state, called “sol-gel transition” or “gelation”. This process is possible thanks to the creation of a crosslinked network between hydrophilic matrix molecules [15]. Different methods are utilized to obtain a hydrogel, but chemical and physical crosslinking are the two more represented approaches. Figure 2 summarizes the different physical and chemical factors.

Physical characterization of the hydrogel involves several properties such as viscosity, degradation rate, injectability and swelling ratio. Depending on the hydrogel designed application these parameters will need to be differently controlled for the material development. Additionally, many of the physical parameters considered in preparing the material can influence one another, for example, crosslink ratio and pH variations influence the swelling capacity, which can be modulated depending on the desired hydrogel application [17,18,19]. Moreover, all these mechanical properties of hydrogels may also have an influence on the hydrogel angiogenic potential [20].

Natural hydrogels function is to mimic the extracellular matrix thanks to the biocompatibility, the biodegradability, and the ability to not cause immune or toxic reaction. It is possible to combine them with synthetic materials in the attempt to reduce the weak mechanical properties, the poor stability and the rapid degradation which represent their mainly limitations. Taken all together these properties indicate the hydrogels as biomaterials ideal for applications in drug delivery technologies, cell encapsulation, contact lenses, scaffolds for tissue engineering, regenerative medicine applications [3,16,21], and in the context of angiogenesis, they are also used as 3D cell culture models to better understand the complex mechanisms underlying this process [22].

## 5. Angiogenic Potential in Natural Hydrogels

**Figure 3:**
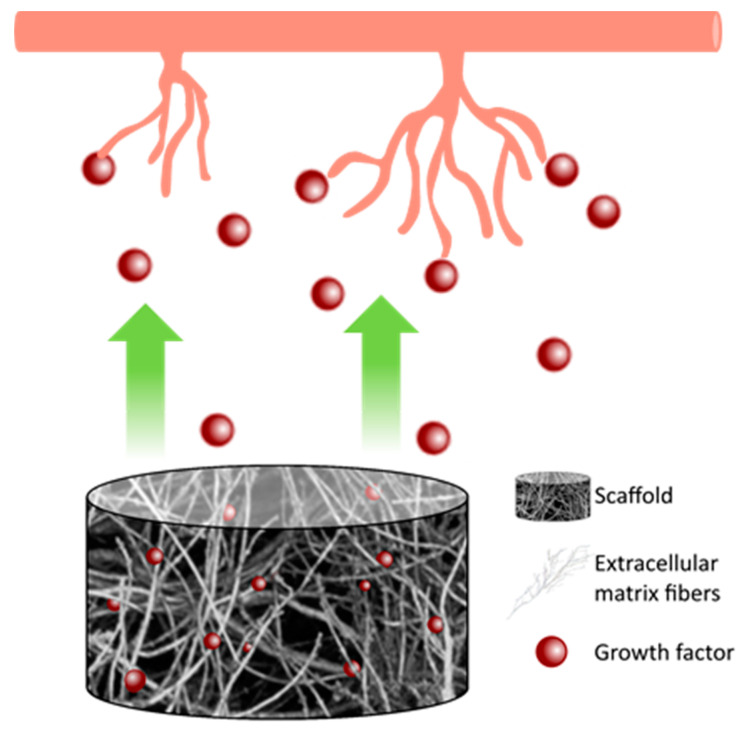
Hydrogel’s release of growth factors stimulates new capillary formation.

Hydrogels derived from natural sources can be used as basic elements of a proangiogenic system. Firstly, the general advantage in using natural hydrogels is the chemical composition similar to the native ECM and the presence of bioactive molecules that gives the material characteristics as an extraordinary biocompatibility while avoiding immune reactions and promoting cell proliferation and differentiation. Moreover, the capability to release GFs, the porosity of natural hydrogels which allows for new capillary network formation, and in some cases, specific chemical compositions, such as collagen and hyaluronic acid (HA), gives them angiogenic potential. Figure 3 represents this process.

On the other hand, rapid degradation, poor stability, and low mechanical strength represent some of the main limits to a wide use in tissue engineering. These problems can be partially solved using synthetic hydrogels because they have better mechanical and biochemical properties. Some examples of this type of hydrogels are poly(ethylene glycol) (PEG), poly(acrylic acid) (PAA), poly(vinyl alcohol) (PVA), and polyacrylamide (PAAm). However, they are not biocompatible and degradable, indeed usually they are used along with natural hydrogels to obtain a co-polymeric hydrogel presenting adequate characteristics [23]. Examples of novel hybrid hydrogels are: a hydrogel created using decellularized tissues, which represent the bioactive component of the hydrogel, mixed with alginate and PVA [24], another example is crosslinking PEG with hyaluronic acid [25], in both examples the synthetic counterpart confers mechanical stability and strength.

Because they all have distinctive characteristics to better suit the final application to achieve different purposes it is possible to choose between various types of hydrogels. Natural hydrogels are usually classified in three main categories: (i) polysaccharides-based, (ii) protein-based and (iii) derived from cellularized tissues [16,21,26,27].

### 5.1. Polysaccharide-Based Hydrogels

Glycosidic bonds link repeated monosaccharide units that create a long carbohydrate molecule called polysaccharide. They are easily available, in fact they represent the most abundant biomolecules in nature which makes them promising biomaterials for several applications including drug delivery, encapsulation of cells, and releasing GFs [28,29].

#### 5.1.1. Glycosaminoglycans

GAGs are long linear polysaccharides. Their structure comprises repeated disaccharide units which can be sulfated, such as heparin, heparan sulfate, keratan sulfate and chondroitin sulfate or nonsulfated, as HA [27]. Depending on the type, the functions of GAGs can change, however they generally affect cell migration, survival and signaling. These functions of GAGs are essential also in the process of angiogenesis indeed, as they can bond VEGF and FGF [30].

Composed by repeated disaccharide units consisting of N-acetylglucosamine and D-glucuronic acid, HA is a linear high molecular weight (about 10^7^ kDa) non-sulfated GAG present in the ECM. Its role consists of a space filler, as a lubricant and helps the processes of wound healing, angiogenesis, and signal transduction [21,27]. HA is characterized by a high biodegradability, biocompatibility, high viscoelasticity and hydrophilia, moreover, it can bind water forming hydrogen bonds with the solvent [31].

HA enzymatic degradation produces fragments of the molecule, called hyaluronan oligosaccharides, of less than 10 disaccharide units, that have shown to promote angiogenesis and wound healing [32,33]. However, since native HA is vulnerable to degradation by hyaluronidase or reactive oxygen species, it is necessary to chemically modify the hydrogel [27], because otherwise the degradation time of the HA scaffold will be faster than the regeneration of the tissue. Modifications can be different: β-cyclodextrin-modified HA (CD-HA) [34,35], adamantane-modified HA (Ad-HA), acrylated HA (AHA) [36,37], dextran-HA (Dex-g-HA) [38], catechol-HA (CA-HA) [29] and methacrylate-modified HA (HAM) [29,39]. The difference in the modifications gives the hydrogel different abilities, for instance, CA-HA hydrogel used in a mouse model of hindlimb ischemia allows the arousing of capillaries and arterioles [29]. Instead, CD-HA/Ad-HA enhance cell retention at the hypoxic border zone of the ischemic myocardium [39]. Generally, this group of hydrogels stimulates angiogenesis because they can incorporate GFs (PDGF, VEGF and FGF) [26,39,40] and encapsulate cells for creating capillary-like structures [29]. Furthermore, to regulate and prolong the release rate of growth factors, heparin can be included into HA hydrogels through the method of heparinization. In fact, heparin is a sulfated GAG that can covalently bind various angiogenic GFs and has the ability to sequester them in the ECM [26,40]. HA also inhibits platelet adhesion and aggregation, and stimulates angiogenesis which makes it suitable for vascular applications [41]. Lastly, HA hydrogels have another important function: they allow adhesion and degradation of endothelial cells when they are crosslinked with MMP-sensitive peptides [42].

#### 5.1.2. Alginate

Alginate is an anionic copolymer derived from brown seaweed containing blocks of 1,4-chain mannitol (M) and L-guluronic acid (G) residues. The G block forms a rapid and reversible crosslink in presence of calcium ions that gives the hydrogel stronger mechanical properties compared to other natural derived hydrogels. If the G block content increases, there will be also an improvement of mechanical abilities. For this reason, alginate is one of the most popular and attractive hydrogels used in fiber-based technologies or as biomaterial for the delivery of therapeutic factors [43]. Physical crosslinking ability, good biocompatibility, non-toxicity, and high viscoelasticity are the main properties of alginate hydrogels. Unfortunately, poor stability, poor cell adhesion, and low mechanical strength represent the major limitations [4,21].

Alginate hydrogels are promising delivery system, for instance they can control and deliver VEGF, PDGF and FGF. Similarly, to the HA hydrogels, alginate hydrogels can be heparinized in order to control the release of GFs [26]. Also, pure alginate gels have the ability to deliver GFs, however they showed a low controlled degradability that is an important limitation in studies “in vivo” [42].

Other molecules that can recruit vascular progenitor cells and induce angiogenesis can be encapsulated in alginate hydrogels. An example is the phospholipid sphingosine-1-phosphate (S1P). For the release of S1P from the alginate hydrogel, a composite alginate-chitosan hydrogel can be used by changing the content of chitosan, the release rate can be controlled [44]. Platelet-rich plasma (PRP) can be incorporated. PRP is a plasma fraction containing several GFs, including VEGF and PDGF which can recruit stem cells and induce angiogenesis. A PRP-alginate-based bioink has been developed for 3D bioprinting scaffolds to elute GFs [43].

A pH-responsive Ca-alginate hydrogel loaded with protamine nanoparticles and hyaluronan oligosaccharides can be used to treat diabetic wounds, which are chronic wounds, and represent a persistent and severe complication of diabetes. These hydrogels regulate antibacterial and neovascularization activities promoting the healing of the wound. In fact, several studies conclude that if the pH becomes more alkaline, there will be an acceleration of the bacterial colonization and biofilm formation, thus prolonging the inflammatory phase and impairing the formation of blood vessels. In addition, because it acts as a cationic antimicrobial peptide, protamine works against a wide range of bacteria causing general disruptions to prokaryotic cells envelope, meanwhile the secretion stimulated by VEGF and the acceleration of the wound healing is a consequence of the addition of hyaluronan oligosaccharides in the hydrogel. Thus, pH responsive Ca-alginate hydrogel loaded with protamine nanoparticles and hyaluronan oligosaccharides enhances endothelial cell capillary-like formation and increases cells in wound healing [45]. Likewise, several studies evidence good results for diabetes mellitus type 1 treatment using alginate hydrogel combined to VEGF for islet encapsulation. This is just another example of how alginate hydrogels can be used as a GFs delivery system [46].

#### 5.1.3. Chitosan

Chitosan is a linear polysaccharide made of N-acetyl-D-glucosamine units, derived from the natural polymer chitin by partial deacetylation. These hydrogels present several advantages such as a good biocompatibility and biodegradability, antibacterial properties, an easy way of controlling degradation, and the possibility of undergoing a sterilization process. On the other hand, inadequate mechanical properties characterize chitosan hydrogels, although it is possible to fix this problem by adding chemical groups or by gelatin crosslinking the hydrogels [21,26].

Moreover, chitosan is commonly used in the fabrication of hydrogels for application in drug delivery and, particularly in wound healing [47]. In this context chitosan hydrogels have also shown potential in in vivo studies, where their application promoted wound closure, ECM remodeling and angiogenesis [48,49].

As the alginate, chitosan hydrogels release S1P as an angiogenic stimulus [50].A second application consists in using chitosan hydrogels crosslinked with PVA to develop a NO releasing hydrogel, that is another necessary factor for the proliferation and migration of endothelial cells. It has been demonstrated that the hydrogel enhances angiogenesis but the molecular mechanisms behind that need to be further investigated [51]. 

### 5.2. Protein-Based Hydrogels

Due to their high biocompatibility and bioactivity, it is common to use proteins-based hydrogels in tissue engineering. Proteins used in hydrogel formation are mainly derived from ECM, such as collagen, or anyhow derived from biological sources, such as fibrin, precisely because these proteins naturally enhance cell adhesion and proliferation [52]. 

#### 5.2.1. Collagen

The most abundant protein in ECM is collagen, which is widely found in tissues such as skin, cartilage, blood vessels, teeth, bones, and tendons [21]. There are 29 types of collagen but collagen type I, II and III are the most represented in the human body and type I is the most used natural scaffold in tissue engineering research, as it is the major protein component of ECM of connective tissues such as skin, bone, tendons and ligaments, meanwhile type II collagen is mainly found in hyaline cartilage and type III collagen is found in elastic vascular tissue [53,54]. The structure can be divided in four levels of organization; at the beginning it is composed by a tripeptide sequence, until the final structure, which is a three-polypeptide chain, snagged to form a rope structure with three strands. The advantages are extremely numerous, such as biocompatibility, biodegradability, low antigenicity, and low inflammatory response. On the other hand, unmodified collagen hydrogels are weak scaffolds and create degradation products which are composed by amino acids generated by collagenases and metalloproteases. These degradation products activate the coagulation cascade and show a thrombogenic potential [16,27].

The applications are mainly related to the ability to mimic the ECM, to allow cells adhesion and to deliver GFs. It is possible to create microfluidic tubes inside the hydrogel where proangiogenic factors and/or endothelial cells are used to induce angiogenesis [37]. Moreover, collagen hydrogels allow the formation of 3D microcapillary networks by endothelial and perivascular cells [39]. Collagen hydrogels were also used as 3D culture models to study angiogenesis pathways, such as Notch signaling [55], and new genes interacting with Notch and VEGF signaling [56].

#### 5.2.2. Fibrin

Fibrinogen is a large glycoprotein present in blood plasma. It plays a role in hemostasis, fibrinolysis, inflammatory response, neoplasia, and wound healing. During these processes, fibrinogen is converted into fibrin by the action of an enzyme named thrombin. Fibrin hydrogels are formed through the polymerization of fibrinogen with thrombin and calcium ions through physical interactions. Moreover, the biggest advantage is the opportunity to extract the fibrin from the patient’s blood, in this way the immune and potential inflammatory responses can be overcome. Unluckily, the fast degradation kinesis in vivo, the poor mechanical properties, the narrow ability to control the matrix rigidity and the low elasticity are the main disadvantages [26,39,42,57].

Fibrin hydrogels have been studied for many reasons mainly because of the intrinsic angiogenic abilities, secondly because of the cells/GFs delivery applications and lastly for being an artificial microenvironment that greatly mimics the native ECMs [37,57].

GFs such as VEGF and FGF can be released by fibrin hydrogels, however, the result is usually uncontrollable and lasts at maximum 24 h. The techniques to overcome this problem are numerous and they can depend on the type of growth factor. Concerning VEGF, a covalent VEGF-modified fibrin gel can be created with an engineered variant of the factor that can covalently bind fibrin via trans glutamination. Using this modified hydrogel in vivo, the nearby cells can repopulate and degrade the matrix, inducing the hydrogel carrier degradation that allows the release of VEGF. Another method to control the releasing time is to bind heparin into the gels. In HA, alginate and gelatin hydrogels, heparin has the ability to bind VEGF and FGF to reduce and control the release rate [42,58]. Furthermore, fibrin hydrogels are perfect materials for 3D creation of blood vessel capillaries because they are capable of accommodating endothelial cells and mesenchymal cells [39,40] and also thanks to hydrogel’s ability to release VEGF and PDGF [58].

#### 5.2.3. Gelatin

Gelatin is a polymer obtained from the hydrolysis of collagen. Containing many arginine-glycine-aspartic acid sequences, gelatin hydrogels allow cell adhesion and improve matrix metalloproteinases abilities. Moreover, they possess a good biocompatibility, biodegradability, low immunogenicity, and cell affinity. On the other hand, low thermal stability and poor mechanical strength are the two main limitations [21,59].

As other hydrogels, gelatin based ones can release different molecules, such as the stromal derived factor-1 (SDF-1) [60]. Because it is able to mobilize endothelial cells and pro-angiogenic bone marrow derived cells, this factor acts in the process of angiogenic healing. The release of SDF-1 from the hydrogel is controlled by the degradation rate and by cells invasion [40]. In fact, gelatin hydrogels can be enzymatically degraded and it is possible to control the range of degradation that can vary from a few days to several months [61].

Moreover, gelatin-based hydrogels also guarantee the release and the bioactivity of VEGF [62]. To achieve a better binding between VEGF and the hydrogel, heparin can be added [63]. Moreover, several studies demonstrate that gelatin hydrogels influence the secretion of VEGF and MMPs from the endothelial cells. For instance, by applying endogenous electrical fields on the gels, the cells are stimulated and release the enzymes and the GF [64].

Likewise, methacrylate gelatin (GelMA) which is typically created by modified extracellular matrix comprising of methacrylate groups added to the amine-containing side groups of the natural gelatin [65], is a potential scaffold for the release of GFs thanks to a low antigenicity and a better solubility [59]. For instance, VEGF can be delivered by GelMA hydrogel to promote the growth of the endothelium [66]. In addition, this hydrogel can also be used for 3D printing of tube-like structures [16].

Another important application of gelatin hydrogel indicates the ability to induce a local hypoxic environment or stimulate hypoxia-inducible factor-1 (HIF-1). A hypoxic microenvironment promotes the formation of vascular system and the angiogenesis process [40,61]. A way to create these conditions consists in using laccase, which is an enzyme with the ability to fully consume oxygen [40]. Another method involves the use of deferoxamine (DFO). In fact, with DFO the level of hypoxia-inducible factor-1 (HIF-1) and VEGF significantly increases compared to the gel without DFO. For this reason, the expression of angiogenesis-related genes increases with DFO addition [61].

### 5.3. Hydrogels Derived from Decellularized Tissues

It is well known that the role of the ECM is essential for a tissue because it can maintain the homeostasis and it can influence many processes, such as the regulation of angiogenesis and cell adhesion. For this reason, the goal of the decellularization consists in achieving sufficient cells and nucleic acids removal from the source tissue while preserving the ECM structure and composition [67,68]. 

**Figure 4:**
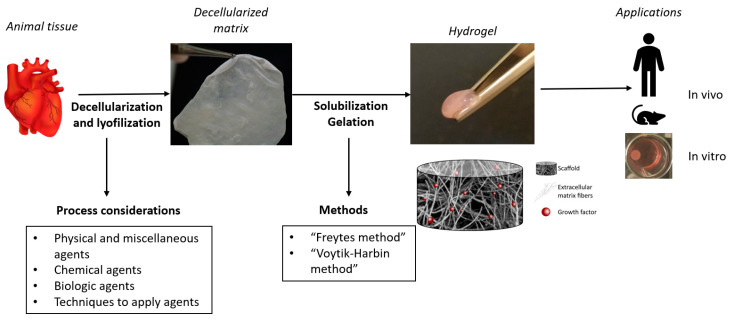
Decellularized ECM hydrogel’s process obtention.

**Figure 5:**
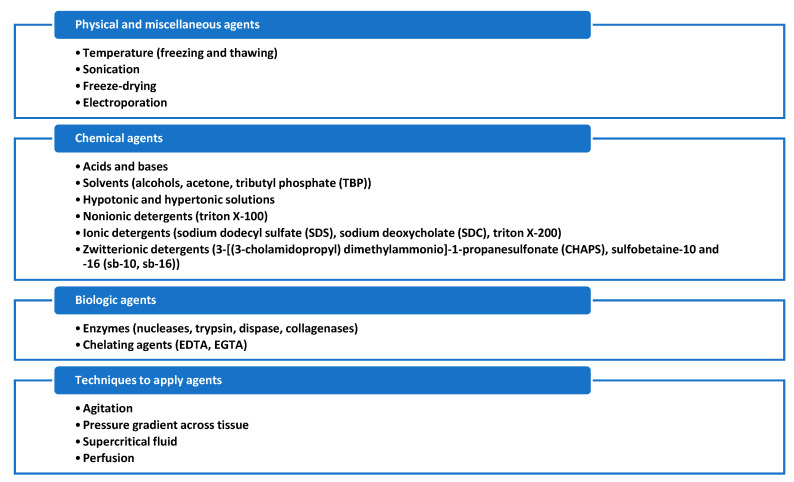
Summary of the different decellularization agents and techniques [69,70,71].

The process to obtain a decellularized hydrogel is schematized in Figure 4. The first step is to decellularize the native tissue. In this context, specific tissue, and organ characteristics, including cell and matrix density, tissue shape, thickness and geometrics should be considered to process with the optimal method of decellularization. To preserve both matrix’s composition and biological activity, the decellularization method should be rationally selected and scientifically justified for each matrix or organ [69]. A crucial point is not to use the optimal physical force exposure only, but also to choose correctly the non-physiologic chemical and biologic agents such as enzymes and detergents. Figure 5 contains the highlights of decellularization agents.

Following the matrix decellularization and lyophilization the second step concerns the solubilization of the ECM material into monomeric protein components. The commonest method used is called “ECM digestion”, and it consists of pepsin digestion in acid solution using hydrochloric acid as long as necessary to cleave the telopeptide bonds of the collagen triple helix structure to unravel collagen fibril aggregates [72]. On the other hand “Voytik-Harbin method” uses acetic acid instead of hydrochloric acid to obtain a suitable medium for the enzyme [73]. As summarized by Saldin et al., different solubilization times will produce different molecules which possess bioactive properties and it can be tailored for each clinical application [74]. 

Due to the ability to maintain the major components of the ECM such as the collagen, the GAGs, and the elastin fibers, the ECM scaffolds can promote cell growth, differentiation, proliferation, migration, and angiogenesis [2]. Indeed, in comparison to the other types of hydrogels, this one also manages to keep the physiological ratio between all the ECM components. Moreover, these hydrogels are used as a 3D culturing model that allow the cell adhesion, becoming a better system to simulate a real tissue [68]. Additionally, Wassenaar et al. suggest the effect of the ECM hydrogel in the blood vessel formation and the macrophages’ attraction through endothelial progenitor cells recruitment [75].

## 6. Conclusions

The goal of this review has been to underline the role of natural hydrogels, with particular interest to the angiogenic potential. A literature review of current studies shows that because the angiogenic process is hard to replicate due to the multiple factors involved, researchers simplify this process by focusing mainly on the behaviors of GFs and cells in the hydrogels. All the hydrogels considered in this review have been tested for factors and molecules involved in angiogenesis, such as VEGF, PDGF, FGF, S1P, and SDF-1. Most of the time the results are promising, however sometimes it is necessary to bind other molecules to the hydrogel in other to improve the ability to deliver these factors. In addition, several studies demonstrate that cells adhesion is achievable and, when combined with GFs, capillary-like structures can be obtained in the hydrogels. The different studies considered in this review highlight the angiogenic potential of these biomaterials, which differs in every type of material used to obtain the hydrogel, which aside from being able to release GF, thanks to their chemical composition and porosity, enable the formation of new capillary networks. Moreover, the combination between different materials is also considered to improve the angiogenic properties and to overcome the limitations given by the material. Results are encouraging and prove that natural hydrogels do have a potential in enhancing the angiogenic process.

However, natural hydrogels still present some limitations. As an example, they possess low mechanical properties and poor stability. For these reasons it will be necessary to further investigate the possible methods to achieve adequate solutions. Thanks to new technologies, it will be possible to improve the abilities of these materials to obtain hydrogels that will mimic native tissues more accurately than ever before.
